# Artificial intelligence as decision support for adolescent depression and anxiety: a mini review of clinical utility, safety, and implementation

**DOI:** 10.3389/fpsyt.2026.1910713

**Published:** 2026-08-13

**Authors:** Xinyue Zhou, Lei Geng

**Affiliations:** 1 Copenhagen Business School, Copenhagen, Denmark; 2 Department of Psychiatry, Wuhan Mental Health Center, Wuhan, Hubei, China

**Keywords:** adolescent mental health, anxiety, artificial intelligence, clinical decision support, depression, digital phenotyping, large language models, risk prediction

## Abstract

Adolescent depression and anxiety are common, heterogeneous, and often recognized late. Young people may present with sleep disturbance, irritability, somatic complaints, school refusal, social withdrawal, attentional difficulties, or functional decline before they meet clear diagnostic thresholds. Artificial intelligence has been proposed as a way to improve early identification, monitoring, triage, and treatment planning. However, predictive accuracy alone does not establish clinical value. This Mini Review examines what artificial intelligence can realistically add to the care of adolescents with depression and anxiety, with emphasis on decision support rather than automated diagnosis. Current evidence suggests potential value in prospective risk prediction, digital phenotyping, measurement-based monitoring, conversational interfaces, and early detection of poor treatment response. These tools may help clinicians identify young people who need closer follow-up, structured assessment, safety planning, or treatment adjustment. However, the evidence remains limited by retrospective designs, selected samples, inconsistent outcome definitions, weak external validation, limited calibration, uncertain incremental value over standard clinical assessment, and insufficient testing in real workflows. Digital and conversational tools also raise concerns about privacy, consent, safeguarding, equity, and over-monitoring. Clinically useful artificial intelligence should therefore be evaluated by whether it improves concrete decisions: who is assessed earlier, monitored more closely, escalated for safety concerns, or offered a different treatment pathway. Future research should prioritize measurement precision, cultural transportability, realistic comparators, and evidence that model-guided care improves outcomes for young people.

## 1 Introduction

Adolescent depression and anxiety are major clinical and public-health problems because they often emerge during a developmental period when symptoms, identity, family relationships, school functioning, peer life, and help-seeking behavior are all changing. Nearly half of all mental disorders begin before age 18, which makes adolescence a critical period for early recognition and intervention (1). Updated Global Burden of Disease estimates also show that mental disorders remain a large and unevenly distributed global burden, with disability-adjusted life-year rates peaking in the 15-19-year age group (2). Yet the clinical signal is often indirect. Adolescents may not initially present with a clear complaint of low mood or excessive worry. Instead, they may show sleep disturbance, irritability, somatic symptoms, school refusal, attentional problems, academic decline, social withdrawal, family conflict, or functional deterioration (3).

This complexity is not simply a diagnostic inconvenience. Anxiety and depression in young people frequently develop through transdiagnostic pathways involving negative affect, threat sensitivity, emotion dysregulation, cognitive control, interpersonal stress, and ecological adversity (4). Broad transdiagnostic frameworks must still be used carefully, because they can understate development, clinical context, comorbidity, and real-world service constraints (5). The practical problem is therefore not only whether an adolescent meets criteria for a depressive or anxiety disorder. Clinicians also need to know who is likely to deteriorate, develop persistent symptoms, engage in self-harm, disengage from care, or respond poorly to first-line treatment.

Artificial intelligence and machine learning have been proposed as tools to address this uncertainty. In principle, these methods can combine clinical symptoms, developmental history, family context, school functioning, digital behavior, neurobiological measures, and treatment data to identify patterns that may not be visible during a single clinical encounter (6, 7). Recent adolescent-focused reviews also show that AI applications now cover risk prediction, personalized support, digital intervention, clinical management, and access expansion (8). In practice, psychiatric prediction literature has repeatedly shown that technical performance does not automatically translate into clinical usefulness. Many prediction models remain limited by selected samples, retrospective design, poor transportability, weak external validation, incomplete calibration, and uncertain clinical consequences (9).

This distinction is especially important in adolescent mental health. A model that distinguishes diagnosed depression from healthy controls may have little value for a school counselor, primary-care clinician, or child and adolescent mental health service deciding whether a young person with insomnia, irritability, and declining attendance needs urgent assessment. Similarly, large language models and conversational agents should be evaluated not only as information tools, but also as possible triage, monitoring, and escalation interfaces that require developmental safeguards, crisis protocols, and clinician oversight (10–13). This Mini Review therefore focuses on clinical utility, safety boundaries, and implementation requirements rather than exhaustive coverage of algorithms. Literature was selected narratively to support a focused discussion of artificial intelligence, digital phenotyping, prediction models, treatment monitoring, neurobiological stratification, and implementation in adolescent depression, anxiety, and broader internalizing problems.

## 2 Near-term clinical roles of artificial intelligence

### 2.1 Earlier identification, triage, and risk prediction

The most defensible near-term use of artificial intelligence in adolescent depression and anxiety is not automated diagnosis, but earlier identification of young people who may need closer assessment or follow-up. This requires a shift from case-control classification to prospective prediction. The relevant target is not ‘depressed versus healthy’, but future onset, persistence, deterioration, self-harm risk, or functional decline.

Several studies have moved in this direction. Machine-learning studies have predicted anxiety onset in adolescents who were non-anxious at baseline, depression and anxiety from multi-wave longitudinal data, and adolescent depression from prenatal and childhood information (14–16). Large developmental cohorts such as the Adolescent Brain Cognitive Development Study also provide longitudinal data across mental health, cognition, family environment, substance exposure, physical health, and neuroimaging (17–20). A recent multitask deep-learning system also used spontaneous textual expressions from Chinese adolescents to estimate PHQ-9 and GAD-7 severity, illustrating that AI screening may become more linguistically and culturally specific (21). These studies are important because they ask a more clinically relevant question than diagnostic classification: which young people may need future clinical attention?

Real-world data may improve scalability but also introduce bias. Electronic health records and social determinants of health can help identify adolescent depression and anxiety, but they may also encode access to care, documentation practices, school referral patterns, and family help-seeking behavior (22). Machine-learning studies using electronic health records have shown potential for suicide-risk prediction in children and adolescents (23, 24), but recent reviews still report limitations in validation, bias assessment, outcome definition, and implementation (25, 26). A risk estimate is useful only if it changes what happens next: earlier review, structured assessment, family contact, school liaison, safety planning, or referral.

### 2.2 Monitoring between clinical encounters and conversational interfaces

Adolescent depression and anxiety fluctuate with sleep, school pressure, family conflict, peer relationships, digital life, treatment exposure, and developmental transitions. A single appointment may miss clinically important deterioration. Digital phenotyping has therefore attracted interest because smartphones and wearable devices can capture sleep, activity, mobility, communication rhythms, and behavioral regularity outside the clinic (27).

Early studies suggest that mobile and wearable sensor data can contribute to depression assessment in adolescents, but the evidence remains exploratory (28). Reviews of smartphone sensing in young people show heterogeneity in measures, short follow-up, inconsistent reporting, and limited evidence that passive data improve care decisions (29). A recent feasibility study integrated active self-reports and passive smartphone data to predict mental-health risk in school-going adolescents, showing the promise of digital phenotyping but also the need to specify the target construct, sensor window, reference standard, and clinical response (30). The central question is not whether a device can detect behavioral change. The question is whether that change can be interpreted safely and acted on appropriately.

Digital phenotyping is most defensible when embedded within measurement-based care. Measurement-based care uses repeated standardized measures to guide treatment decisions and has an emerging evidence base in child and adolescent psychiatry (31). In adolescent depression and pediatric anxiety, it can support systematic review of symptoms, suicidality, functioning, adverse effects, and treatment progress (32, 33). Artificial intelligence may strengthen this approach by identifying slower-than-expected improvement, worsening sleep or activity patterns, disagreement between adolescent and parent reports, or early signs of relapse. Conversational agents and large language models may also support psychoeducation, structured symptom capture, appointment preparation, and follow-up prompts, but they should not become autonomous diagnosis, crisis management, or continuous surveillance without a defined clinical purpose (10–13). Recent US surveys suggest that adolescents and young adults already use generative AI and AI chatbots for mental-health advice, often outside clinician awareness (34, 35). This makes chatbot use itself a possible assessment and safety-planning topic, not only a future technology question.

### 2.3 Treatment planning and early non-response

Treatment selection is a high-value use case because delayed response has real consequences. Adolescents may receive psychotherapy, medication, combined care, school-based support, family intervention, or stepped care, but treatment decisions are constrained by availability, severity, comorbidity, safety concerns, family preference, and local service capacity. Machine learning may help identify young people who are unlikely to respond to a first intervention and who may need earlier treatment adjustment.

Evidence is emerging but not yet implementation-ready. Studies have reported machine-learning-guided early prediction of acute outcomes in depressed children and adolescents treated with antidepressants, remission after cognitive behavioral therapy for childhood anxiety disorders, and treatment outcome across concatenated youth anxiety treatment datasets (36–38). A broader systematic review and meta-analysis of treatment-response prediction in emotional disorders found promise but also emphasized the need for stronger validation and clinical integration (39). The key issue is actionability. If a model predicts poor response, the clinical response should be predefined: increasing treatment intensity, reviewing adherence, adding parent or school work, monitoring safety more closely, or seeking specialist input. Without an action pathway, prediction is information without clinical consequence.

## 3 Why predictive accuracy is not clinical utility

### 3.1 Validation, calibration, thresholds, and base rates

Artificial intelligence models for adolescent depression and anxiety often perform better inside research datasets than they are likely to perform in ordinary services. Internal validation cannot show whether a model will work across schools, clinics, emergency departments, primary-care systems, countries, ethnic groups, socioeconomic contexts, or digital environments. This is a central concern in psychiatry prediction models generally (9). Reporting and bias-assessment standards have improved. TRIPOD+AI clarifies how prediction models using regression or machine learning should be reported, while PROBAST+AI provides a framework for assessing risk of bias and applicability (40, 41). For studies that proceed to clinical evaluation, CONSORT-AI, SPIRIT-AI, and DECIDE-AI are also relevant, and imaging-specific work may also require checklist-based reporting such as CLAIM (42–45).

Discrimination is not enough. A model may have an acceptable area under the curve while still producing poorly calibrated risk estimates. Calibration matters because clinicians need to know whether a predicted risk corresponds to observed risk in similar young people. This is particularly important for low-base-rate outcomes such as suicide attempts or near-term self-harm. Even models with apparently good discrimination can generate many false positives when the outcome is rare. False positives may stigmatize a young person, alarm families, increase surveillance, or divert limited services. False negatives are equally serious because they may delay assessment during escalating risk. Net-benefit approaches can help evaluate whether acting on a model produces more benefit than harm across clinically meaningful thresholds (46).

Thresholds must also match service capacity. A school-based model that identifies 25% of students as high risk may be unusable if there is no pathway for assessment. A clinic model that flags deterioration daily may create alert fatigue. A self-harm model that provides risk scores without a crisis protocol is unsafe. Clinical utility requires a threshold, an action, and a system able to deliver that action.

### 3.2 Comparators, leakage, and transportability

A complex model is not clinically valuable simply because it is complex. It must improve on realistic comparators such as recent symptom severity, prior episodes, school attendance, sleep disturbance, parent or teacher concern, clinician judgment, standard screening tools, measurement-based care, or simple regression models. Reviews of machine learning in mental health and medicine have repeatedly warned that algorithmic sophistication can obscure weak design, poor reporting, and overclaiming (47–49).

Outcome leakage is another risk. Electronic health record models may appear to predict future diagnosis while relying on variables that already reflect current clinical recognition, such as previous service use, medication, referral patterns, or clinician concern. In that case, the model may predict documentation rather than illness. This matters because adolescents with less access to care, lower family advocacy, minority language status, or mistrust of services may be systematically under-recognized. The well-known example of racial bias in a population-health algorithm shows how health-care utilization can become a biased proxy for need (50). For adolescent depression and anxiety, models should therefore be judged by incremental value: if artificial intelligence does not outperform a small set of clinically obvious predictors, it may add burden without improving care.

### 3.3 Digital, conversational, and adolescent-specific risks

Digital mental-health tools are not neutral. Preventive digital interventions for young people have often been limited by variable design quality, incomplete reporting, and uncertain implementation evidence (51). Conversational agents may provide psychoeducation, self-help support, or symptom tracking, and reviews suggest possible benefits for mental health outcomes (10, 11). However, large language models and generative artificial intelligence introduce additional risks because they can produce fluent, persuasive responses without guaranteed clinical reliability (12, 13). Reporting standards are therefore important. The CHART statement for chatbot health-advice studies emphasizes transparent reporting of model identity, version, prompt strategy, query sampling, reference standard, performance evaluation, and harmful, biased, or misleading outputs (52).

These risks are amplified in adolescence. Young people vary in autonomy, insight, crisis vulnerability, digital literacy, and willingness to disclose risk. Consent and assent are complicated by parental responsibility, safeguarding duties, and the adolescent’s need for privacy. Passive sensing may collect sensitive information about sleep, location, movement, communication, school routines, peer contact, and family conflict. In some cases, monitoring may help detect deterioration; in others, it may become intrusive surveillance. Equity also matters. Device ownership, data plans, phone-use rules, language, disability, rurality, ethnicity, socioeconomic status, and health-system access can all affect who is represented in training data and who benefits from deployment. A model that works best for digitally connected, service-engaged adolescents may widen rather than reduce disparities.

## 4 A decision-first implementation framework

### 4.1 Start from the decision, not the algorithm

Artificial intelligence should be developed around clinical decisions. The starting question should be: what decision is difficult, consequential, and currently poorly supported? Examples include whether a young person should receive further assessment, whether follow-up should be brought forward, whether self-harm risk requires same-day review, whether treatment should be intensified, whether school coordination is needed, or whether a waiting-list case should be prioritized.

**Figure 1:**
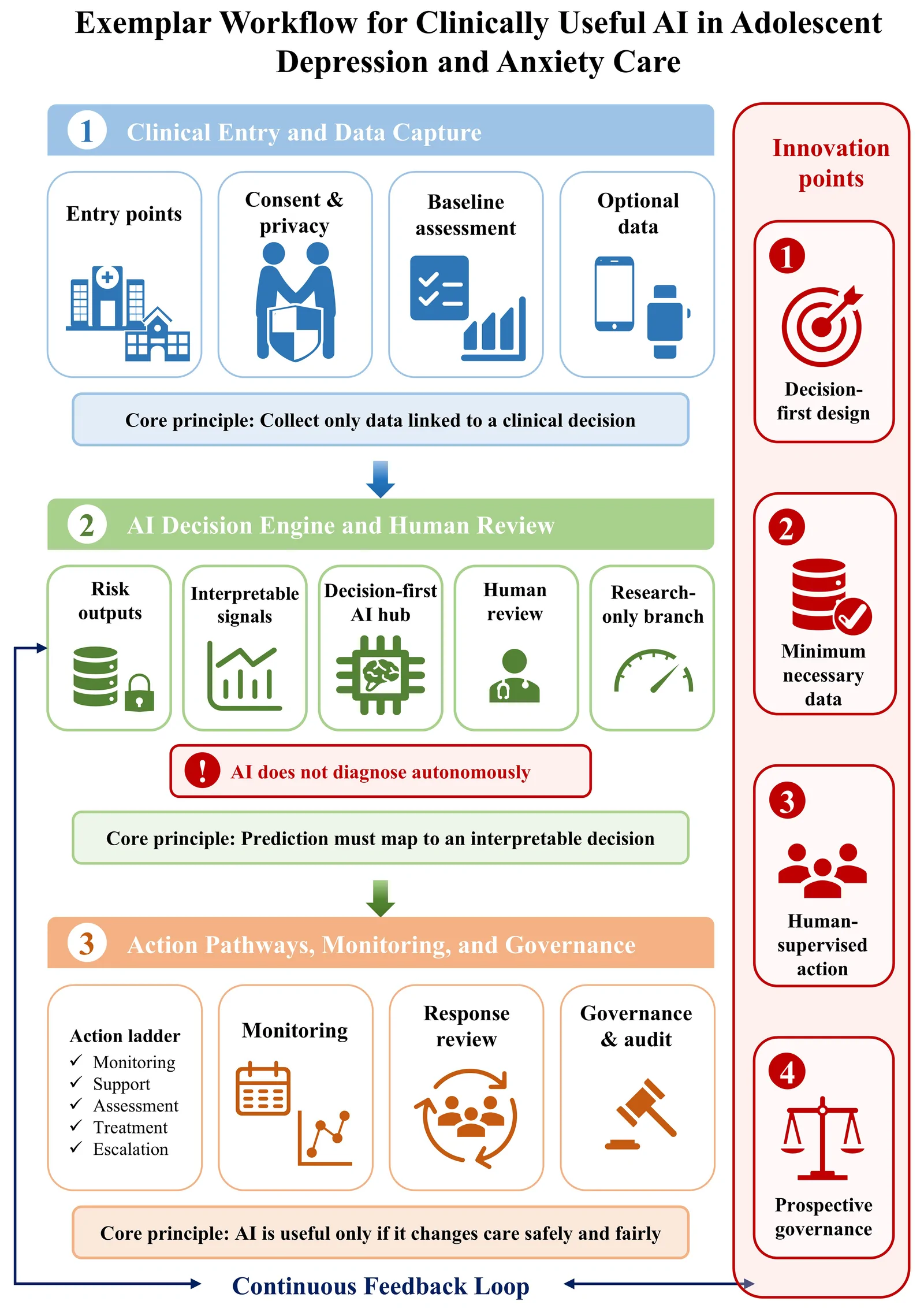
Exemplary workflow for clinically useful artificial intelligence in adolescent depression and anxiety care. The framework connects clinical entry and minimum-necessary data capture with an AI decision engine that produces interpretable outputs for mandatory human review. Model outputs are linked to predefined action pathways, monitoring, response review, governance, and a continuous feedback loop. The workflow emphasizes decision-first design, minimum necessary data, human-supervised action, and prospective governance. AI is intended to support clinical decisions and does not diagnose autonomously.

This decision-first approach aligns with broader concerns about delivering clinical impact with artificial intelligence (53). It also prevents a common error: building a model because data are available rather than because a clinical decision needs support. The minimum standard is not whether the model predicts an outcome. It is whether the model changes a decision in a way that improves safety, access, functioning, or symptoms. **Table 1** summarizes a practical clinical-utility framework, and **Figure 1** illustrates how prediction should connect to human review, action pathways, monitoring, and governance.

**Table 1:** Clinical utility framework for artificial intelligence in adolescent internalizing disorders.

Dimension	Key question	Minimum evidence needed
Clinical task	What decision is the model supposed to support?	Define the use case before model development: screening, triage, monitoring, safety review, treatment adjustment, or mechanism generation.
Target population	Who is the model for?	Report age range, setting, inclusion criteria, symptom severity, comorbidity, and referral context.
Prediction horizon	What time frame is being predicted?	Predefine the outcome window and explain why it matters clinically.
Comparator	What is the model being compared against?	Compare with realistic baselines, including standard rating scales, usual care, or simple clinical rules.
Validation	Does the model work outside the original dataset?	Report external validation, subgroup performance, missing-data handling, and applicability to the intended setting.
Calibration	Are the risk estimates believable?	Report calibration plot, calibration slope and intercept, observed versus predicted risk, and ideally decision-curve or net-benefit analysis.
Actionability	What happens after a high-risk result?	Define the response pathway, responsible clinician, escalation protocol, and service capacity to act.
Interpretability	Can clinicians and families understand the output?	Provide clinician-reviewable explanations and avoid opaque risk scores without clinical meaning.
Safety and equity	What harms could the model create?	Assess bias, subgroup calibration, privacy protection, consent, crisis protocols, and inequitable performance.
Workflow integration	Can this work in real services?	Test usability, alert burden, staff responsibility, workflow fit, escalation pathways, and prospective audit.

AI, artificial intelligence. Clinical utility requires a defined decision, a responsible human reviewer, and a clear action pathway, not only good model performance.

### 4.2 Use interpretable outputs that clinicians can act on

Interpretability should not mean decorative feature-importance plots. In adolescent mental health, an interpretable output should help clinicians understand why a young person has been flagged and what action is appropriate. A useful model might indicate that worsening sleep, school non-attendance, previous anxiety, family stress, and recent symptom escalation contributed to higher risk. An unhelpful model provides an opaque score with no clinical pathway.

Interpretability requirements will differ by task. For triage, clinicians need a small number of reliable reasons for prioritization. For monitoring, they need to know whether change reflects clinically meaningful deterioration or normal fluctuation. For treatment planning, they need to know whether predicted non-response should trigger dose optimization, psychotherapy adjustment, family work, school support, or specialist referral. In all cases, responsibility must remain with qualified professionals.

### 4.3 Keep biomarkers in the correct role

Neuroimaging and biological markers may help explain heterogeneity, but they are not ready for routine individual-level decision-making in adolescent depression and anxiety. Large multimodal datasets, functional-connectivity studies, reward-circuitry research, connectivity biotypes, error-monitoring work, and neurobiological subtyping all illustrate the potential of stratification research (54–64). The limitation is clinical translation: a group-level association is not an individual-level decision tool.

Genetic and molecular approaches should be treated similarly. Genome-wide studies, eQTL, transcriptomic, Mendelian-randomization, colocalization, single-cell genomics, perturbation models, and virtual-cell approaches may support mechanism discovery and drug-repurposing hypotheses (65–74). At present, however, these tools should not be presented as clinical decision aids for adolescents. They may inform stratified psychiatry only when subgroups predict prognosis, treatment response, or care needs better than ordinary clinical assessment (75).

### 4.4 Test implementation, safety, and equity prospectively

The next phase of research should test artificial intelligence within real care pathways. A clinically useful model should specify the target population, prediction horizon, input data, output format, threshold, action pathway, clinician responsibility, escalation process, and audit mechanism. It should report calibration, external validation, subgroup performance, missing-data handling, and net benefit. It should also evaluate whether model-guided care improves outcomes, reduces delay, improves safety, or increases equity.

This agenda is consistent with recent reviews of artificial intelligence in adolescent mental-health care, which identify applications across screening, diagnosis, monitoring, treatment support, and risk prediction but also emphasize the need for stronger evidence and ethical safeguards (8, 76, 77). The field should now move from proof-of-concept models to clinically accountable systems. For adolescent depression and anxiety, the strongest near-term path is not autonomous diagnosis. It is carefully governed decision support embedded in assessment, monitoring, triage, and treatment review.

## 5 Discussion

Artificial intelligence has plausible value for adolescent depression and anxiety, but its most realistic near-term role is decision support rather than autonomous diagnosis. The strongest opportunities are earlier recognition of hidden risk, structured monitoring between clinical encounters, triage support, and early identification of poor treatment response. These applications matter because adolescent symptoms are often fluctuating, indirect, and shaped by family, school, developmental, and cultural contexts.

Current evidence does not yet justify broad clinical deployment. Many models remain limited by retrospective design, selected samples, weak external validation, poor calibration, unclear thresholds, limited comparison with standard care, and insufficient testing in real clinical workflows. Digital and conversational tools also raise specific concerns about privacy, consent, safeguarding, equity, crisis mismanagement, and over-monitoring.

### 5.1 Measurement precision and cultural transportability

A further requirement is measurement precision. In adolescent depression and anxiety, the outcome should not be described only as “mental health risk” or “clinical deterioration”. Future AI studies need to specify the construct being measured, the instrument or reference standard used, the informant source, the prediction horizon, and the clinical action linked to the result. For example, detecting elevated depressive symptoms on a self-report scale, predicting clinician-diagnosed major depression within 12 months, identifying suicidal ideation over the next week, and detecting poor treatment response after four therapy sessions are different tasks. They require different thresholds, acceptable false-positive rates, escalation pathways, and ethical safeguards. Calibration is particularly important because a risk estimate is clinically meaningful only when predicted risk corresponds to observed risk in the intended population. Subgroup calibration is also necessary because symptom expression, disclosure, help-seeking behavior, stigma, school expectations, family involvement, digital access, and service availability differ across cultures and health systems. Therefore, measurement precision is not only a technical issue. It is also a developmental, cultural, and implementation issue. AI should support adolescent mental health care only when the measured target, threshold, comparator, action pathway, and responsible human reviewer are all explicitly defined.

Future studies should begin with clinical decisions rather than algorithms. They should define the target population, prediction horizon, outcome, comparator, action pathway, and safety protocol before model development. They should report calibration and subgroup performance, evaluate net benefit, and test whether model-guided care improves outcomes for young people. In adolescent mental health, a useful artificial-intelligence system is not one that merely predicts risk. It is one that helps services act earlier, more safely, and more fairly.
